# Histopathological and immunohistochemical characterisation of hepatic granulomas in *Leishmania donovani*-infected BALB/c mice: a time-course study

**DOI:** 10.1186/s13071-018-2624-z

**Published:** 2018-01-31

**Authors:** Francisco J. Salguero, Waldo L. Garcia-Jimenez, Isadora Lima, Karin Seifert

**Affiliations:** 1 Department of Pathology and Infectious Diseases, School of Veterinary Medicine, University of Surrey, Guildford, UK; 2 Fundação Oswaldo Cruz, Centro de Pesquisas Gonçalo Moniz, Salvador, Bahia Brazil; 3 Faculty of Infectious and Tropical Diseases, London School of Hygiene & Tropical Medicine, London, UK

**Keywords:** *Leishmania donovani*, Visceral leishmaniasis, Histopathology, Immunohistochemistry, Macrophage, Granuloma

## Abstract

**Background:**

Visceral leishmaniasis (VL) is a neglected tropical disease (NTD), caused by the intracellular protozoan parasites *Leishmania donovani* and *Leishmania infantum*. Symptomatic VL is considered fatal when left untreated. At present, there is no effective vaccine licensed for human use and available chemotherapies have limitations. Understanding the local immune mechanisms required for the control of infection is a key factor for developing effective vaccines and therapeutics.

**Methods:**

We have investigated the development of the typical granulomatous lesions in the liver in experimental VL over time, together with the local immune responses. BALB/c mice were infected intravenously with a dose of 2 × 10^7^ *L. donovani* amastigotes (MHOM/ET/67/HU3) and sacrificed at 15, 35 and 63 days post-infection (dpi). Histopathology and immunohistochemical techniques were used for the detection of *Leishmania* antigen, selected cell types including B and T lymphocytes, macrophages and neutrophils (CD45R-B220+, CD3+, F4/80+ and Ly-6G+) and iNOS.

**Results:**

Granulomatous lesions were identified as early as 15 dpi in the livers of all infected animals. Three categories were used to classify liver granulomas (immature, mature and clear). Clear granulomas were exclusively detected from 35 dpi onwards. Kupffer cells (F4/80+) were predominant in immature granulomas, regardless of the dpi. Nonetheless, the highest expression was found 63 dpi. Positive staining for iNOS was mainly observed in the cytoplasm of fused Kupffer cells and the highest expression observed at 35 dpi. T cells (CD3+) and B cells (CD45R-B220+) were predominant in more advanced granuloma stages, probably related to the establishment of acquired immunity. Neutrophils (Ly-6G+) were predominantly observed in mature granulomas with the highest expression at 15 dpi. Neutrophils were lower in numbers compared to other cell types, particularly at later time points.

**Conclusions:**

Our results reflect the role of macrophages during the early stage of infection and the establishment of a lymphocytic response to control the infection in more advanced stages.

## Background

Leishmaniasis is one of the most prevalent parasitic public health problems worldwide [1, 2]. This term includes cutaneous leishmaniasis (CL), mucocutaneous leishmaniasis [3] and visceral leishmaniasis (VL) [2, 4]. VL is caused by the intracellular protozoan parasites *Leishmania donovani* in Asia and Africa and *Leishmania infantum* in Latin America and the Mediterranean region [5]*.* VL has a high mortality rate if untreated and is estimated to cause 0.2–0.4 million new cases and 20,000–40,000 deaths per year worldwide [1]. Parasites are transmitted by female phlebotomine sandflies to mammalian hosts. Humans are the only known reservoir of *L. donovani*, while canines are the main reservoir for *L. infantum* [6]. In the Mediterranean basin and Latin America, VL is considered a zoonosis caused by *L. infantum.* Northward spread of VL endemic foci in Italy has been reported [7].

At present, there is no vaccine licensed for human use against VL. Limitations of current chemotherapeutic treatments include drug toxicity, long treatment courses, challenging routes of drug administration, drug stability in hot climates and geographical differences in clinical responses to treatment [8, 9].

Understanding the immune mechanisms required for control of infection within the varied tissue microenvironments that contain *Leishmania*-infected macrophages is a key factor for developing effective vaccines and therapeutics. Due to the intrusive techniques required to analyse such responses in human VL patients, current knowledge of host responses in tissues largely derives from experimental animal models, which include mice, hamsters and dogs. The granulomatous pathology of leishmaniasis, across different disease manifestations and in humans and animal hosts, has recently been reviewed [10]. Many of the cellular and molecular components of acquired immunity necessary for the formation, maintenance and effector function of granulomas have been characterised through the use of gene-targeted mice or in vivo administration of neutralizing or depleting monoclonal antibodies [10–12]. Here we characterized the local host and immune response in hepatic tissue from *L. donovani*-infected BALB/c mice over time, using histopathological and immunohistochemical analyses.

## Methods

### Experimental design

A total of 24 BALB/c mice (Charles River, UK) were used for this study. *Leishmania donovani* (MHOM/ET/67/HU3) amastigotes were harvested from the spleen of a Rag-1-knockout (B6) mouse (LSHTM breeding colony, infected > 40 days), re-suspended in RPMI 1640 medium without serum and used for the infection of 18 mice through a 0.2 ml intravenous bolus injection into a tail vein, corresponding to 2 × 10^7^ amastigotes. One group of 6 mice was left uninfected.

Groups of 6 mice were euthanized by cervical dislocation at three different time points, 15 dpi (6 infected mice + 6 uninfected mice), 35 dpi (6 infected mice) and 63 dpi (6 infected mice). Livers were removed and their weights recorded. Impression smears were prepared and fixed in 100% methanol to allow determination of parasite burden and confirm infection. A small piece of liver for each animal was cut off and placed in zinc salt fixative [13] for 16 h and transferred to 70% ethanol for 4 h before processing into paraffin wax. Liver tissue samples were also immersed in 10% neutral buffered formalin solution and processed routinely into paraffin wax.

### Histopathology

Four-μm tissue sections were stained with Hematoxylin and Eosin (H&E) and analysed under light microscopy to identify lesions. The total number of granulomas together with the granuloma size (area) was calculated in a full liver section per animal using a Nikon Eclipse Ni-U microscope and digital image analysis software (Nikon NIS Br, Nikon, Japan). Along with this, granulomas in the liver were classified into three categories according to cellular composition and evolution stage within each time point. Category 1, corresponding to “immature granulomas”, was defined as individual or few fused Kupffer cells without or with few mononuclear cells grouped loosely and harbouring a high number of amastigotes. Category 2 or “mature granulomas” were defined as tightly fused Kupffer cells surrounded by cellular infiltrate, with or without evidence of collagen deposition and with presence of some amastigotes. Category 3 or “clear granulomas” were defined as well formed granulomas with few fused Kupffer cells surrounded by a cellular infiltrate, normally with evidence of collagen deposition and without presence of amastigotes.

### Immunohistochemistry

The immunohistochemical techniques are summarised in Table 1. Formalin or zinc salt fixed liver samples were cut in 4 μm sections, dewaxed and rehydrated at 19 °C, and placed in a fresh solution of 3% hydrogen peroxide in methanol for 15 min to block endogenous peroxidase activity. Samples were then washed in tap water. Epitope demasking was not necessary for zinc salt fixed samples. However, antigen retrieval in formalin fixed samples was achieved with enzymatic digestion, using a solution of 2% proteinase K (Dako, Glostrup, Denmark) in tris-buffered saline (0.05 mol/l Tris-HCl pH 7.5–7.7) or by heat digestion (water-bath, pH 6.0 Dako buffer, 20 min, 95 °C) depending on the primary antibody used (Table 1). Running tap water was then used to wash the tissue sections and the slides were then mounted onto Shandon coverplates (Thermo Fisher Scientific, Runcorn, Chesire, United Kingdom) and loaded into Sequenza® trays (Shandon Scientific). Once mounted, the slides were washed with tris-buffered saline (TBS) (0.85% NaCl, 0.0605% Tris, adjusted to pH 7.5 using 1 M HCl) and then 190 μl of Universal Blocker™ Blocking Buffer in TBS (Thermo Fisher Scientific, Runcorn, Chesire, United Kingdom) was added as blocking agent. After 20 min, 190 μl of the primary antibody was added. The incubation time varied for each primary antibody used. After two washes with TBS buffer, 190 μl of biotinylated link antibody (Table 1) and link block were added, followed by two further buffer washes, 30 min later. Primary and secondary antibody binding was amplified using Ultra-Sensitive ABC Peroxidase Rabbit IgG Staining Kit (Thermo Fisher Scientific, Runcorn, Chesire, United Kingdom) and visualized using the Vector® *NovaRED™* Substrate Kit (Vector Laboratories, Burlingame, California, USA). Unbound conjugate was removed prior to Vector® *NovaRED™* Substrate Kit application with two buffer washes. Slides were then washed in purified water, removed from the coverplates and placed in a rack. Samples were rinsed with tap water for 5 min, before being placed in Mayer’s Haematoxylin counterstain, followed by a further wash in tap water. Finally, sections were dehydrated, cleared and mounted for analysis.

**Table 1:** Antibodies and reagents used for immunohistochemistry

Primary antibody	Antibody type	Dilution	Supplier	Epitope demasking method	Link antibody (dilution)	Buffer
CD3	Rabbit *vs* Human CD3 (polyclonal)	1/500	Dako (A0452) (Glostrup, Denmark)	Proteinase K (Dako)	Goat *vs* Rabbit (1/1000)	TBS
CD45R (B220)	Rat *vs* Mouse CD45R (B220) (monoclonal)	1/100	Invitrogen (RA3-6B2) (Thermo Fisher, Darmstadt, Germany)	pH 6 citrate-buffer (Dako)	Goat *vs* Rat (1/200)	TBS
Ly-6G (Gr1)	Rat *vs* Mouse Ly-6G (Gr1) (monoclonal)	1/25	Invitrogen (RB6-8C5)	pH 6 citrate-buffer (Dako)	Goat *vs* Rat (1/200)	TBS
F4/80	Rat *vs* Mouse F4/80 (monoclonal)	1/100	BioRad (MCA 4976) (Watford, Hertfordshire, UK)	None (ZSF samples)	Goat *vs* Rat (1/200)	TBS
iNOS	Rabbit *vs* Mouse iNOS (polyclonal)	1/500	Merck Millipore (ABN26) (Watford, Hertfordshire, UK)	None (ZSF samples)	Goat *vs* Rabbit (1/1000)	TBS
Anti-Leish	Rabbit *vs* *Leishmania* spp.	1/50	ISCIII (Madrid, Spain)	None	Goat *vs* Rabbit (1/1000)	TBS

### Image analysis

Immunolabelled sections were analysed using light microscopy and digital image analysis (Nikon NIS Br, Nikon Instruments Europe BV, Amsterdam, The Netherlands). The slides were examined with the 40× objective to give a final magnification of 400×, to ascertain the percentage of immunostained area in the lesion. The whole area of the granuloma was selected as region of interest (ROI), and the area with immunohistochemically positive reaction within the ROI was calculated by the software after setting the thresholds. The results are expressed as the percentage of positively immunolabelled area within the total area of the granuloma.

### Statistics

Chi-square test was applied to analyse differences between the number and category of granulomas per time point in the liver. Student’s t-test was used to assess the significance of the differences in the size between granuloma categories.

For IHC, Student’s t-test was performed to compare the mean expression of each immune marker within granuloma categories and time points. Differences were considered significant at *P* < 0.05. The results of immunohistochemical analyses are expressed as group mean ± standard deviation (SD). All analyses were conducted using SPSS 19 software package (SPSS Inc., Chicago, IL, 60606, USA) and GraphPad Prism 7.0 (San Diego, USA).

## Results

### Confirmation of infection

The hepatic parasite burden 15 dpi, expressed in Leishman-Donovan units (LDUs), was 456 ± 65 (group mean ± SD), confirming previously observed levels of infection. Liver weights (group mean ± SD) at 15 dpi, 35 dpi and 63 dpi were 1041 ± 90 mg, 1365 ± 107 mg and 1412 ± 71 mg, respectively.

### Histopathology

**Fig. 1:**
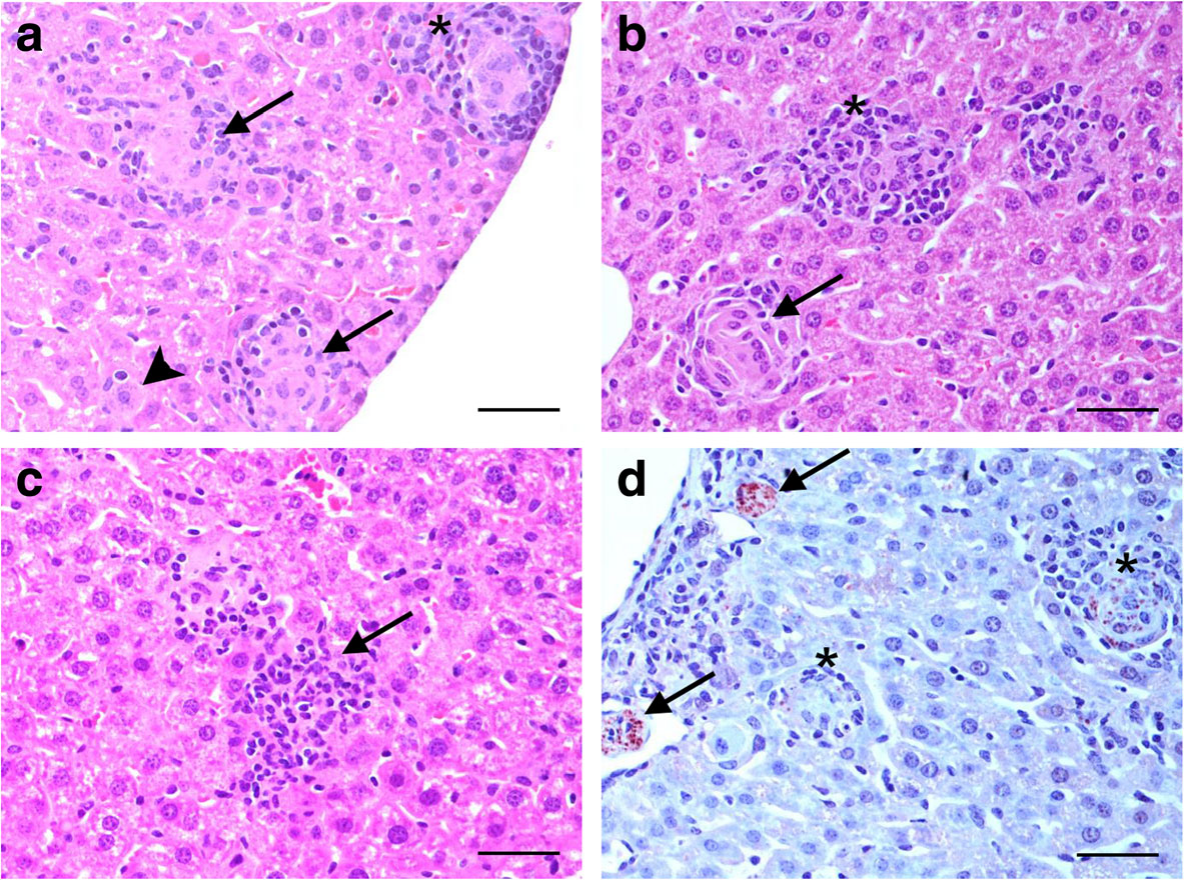
**a** H&E-stained liver section 15 dpi (400×). Kupffer cell harbouring a high amount of amastigotes (arrowhead). Immature granulomas formed by fused macrophages harbouring few amastigotes (arrows). Well-formed mature granuloma with a complete cellular infiltrate (*). **b** H&E-stained liver section 35 dpi (400×). Mature functional granuloma with few fused macrophages harbouring very few amastigotes (*). Clear granuloma without amastigotes and with presence of collagen deposition (arrow). **c** H&E-stained liver section 63 dpi (400×). Clear granuloma composed mainly of lymphocytes (arrow). **d** Immunohistochemical detection of *Leishmania donovani* antigens (400×). Immature granulomas harbouring a high amount of amastigotes (arrows) and mature granulomas with fewer number of amastigotes (*). *Scale-bars*: 100 μm

Granulomas and aggregations of macrophages were present in the livers of all infected animals from 15 dpi onwards (Fig. 1). After the analysis of an entire section of liver for each animal, we characterized (categories) and measured a total of 2467 granulomas from the infected animals. Statistically significant differences (*P* < 0.001) were found in the mean size of granulomas, depending on their category (Table 2). Mature granulomas were larger compared to the other two categories (Immature *vs* mature: *t* = -38.410, *P* < 0.001; Immature *vs* clear: *t* = 4.181, *P* < 0.001 and mature *vs* clear: *t* = 18.669, *P* < 0.001). The granuloma size of each category remained homogeneous between the different time points analysed.

**Table 2:** Mean size of granulomas per category

Granuloma category	Mean size (μm^2^)	Number (*n*)	SD	Error
Immature	30,434.05*	1061	13,471.478	413.578
Mature	66,612.02*	1238	28,030.675	796.660
Clear	25,925.49*	168	9323.050	719.289

*Abbreviation*: *SD* standard deviation

*P < 0.001, when mean size is compared among the three granuloma categories

**Fig. 2:**
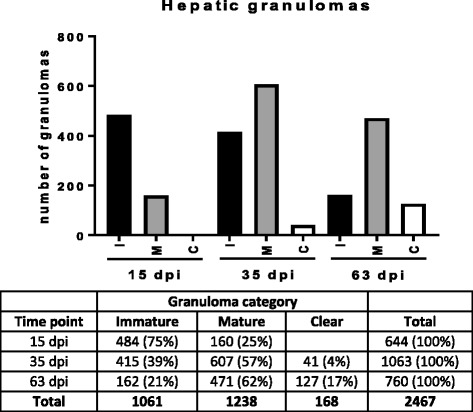
Distribution (total numbers and percentages) of hepatic granulomas induced by *Leishmania donovani* divided into category 1 (immature), category 2 (mature) and category 3 (clear) at three different time points (15 dpi, 35 dpi and 63 dpi)

The number and category of hepatic granulomas varied among the three time points (Fig. 2). At 15 dpi, 75% of granulomas were immature, with no clear granulomas being observed. At 35 dpi, the total number of granulomas increased by 40% compared to 15 dpi. In this case, 57% of granulomas were mature and almost 4% had begun to be resolved (clear granulomas). At 63 dpi, the total number of granulomas was reduced by 30% in comparison with 35 dpi, with 62% of mature granulomas and almost 17% of clear granulomas.

### Immunohistochemistry

#### Detection of *Leishmania* spp. antigen

A specific and strong positive signal was observed for amastigotes inside Kupffer cells in the liver (Fig. 1d).

#### Kupffer cells (F4/80+) and iNOS+ cells

**Fig. 3:**
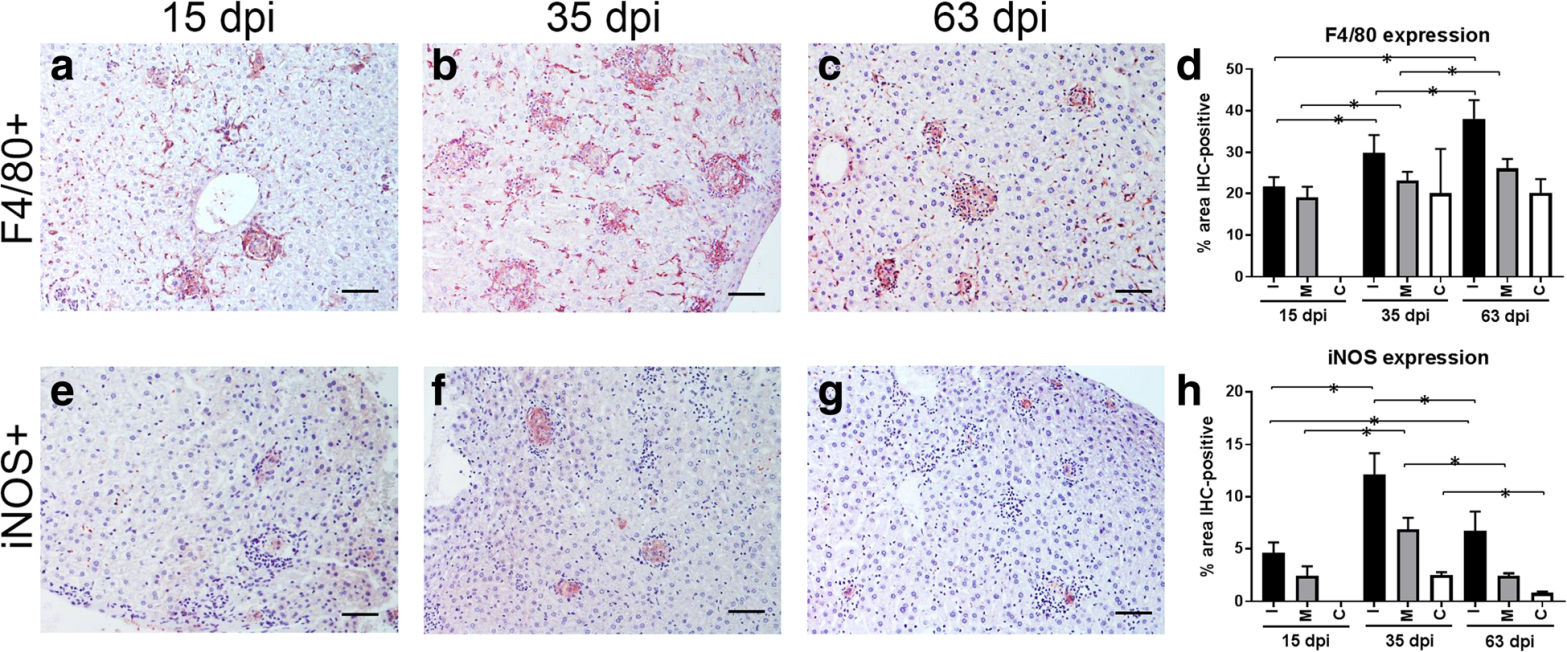
Immunohistochemical detection of macrophages (F4/80+) within hepatic granulomas at 15 dpi (**a**), 35 dpi (**b**) and 63 dpi (**c**). **d** Percentage of immunostained area for F4/80 at 15, 35 and 63 dpi within immature (I), mature (M) and clear (C) granulomas. Immunohistochemical detection of iNOS+ cells at 15 dpi (**e**), 35 dpi (**f**) and 63 dpi (**g**). **h** Percentage of immunostained area for iNOS at 15, 35 and 63 dpi within immature (I), mature (M) and clear (C) granulomas. *P < 0.05. *Scale-bars*: 50 μm

Immunohistochemistry (IHC) for F4/80 was used to locate Kupffer cells in the liver. A decrease in the expression of F4/80 was observed as the granuloma category evolved within the same time point. However, a statistically significant increase in the expression of F4/80 was noted when immature and mature granulomas were compared across the different time points (15 *vs* 35 dpi, immature: *t* = -3.291, *P* < 0.001 and mature: *t* = -2.202, *P* < 0.029; 15 *vs* 63 dpi, immature: *t* = -5.691, *P* < 0.001 and mature: *t* = -4.335, *P* < 0.001) There were no differences in the expression of F4/80 observed for clear granulomas when animals euthanized at 35 and 63 dpi were compared. The highest expression of F4/80 was found in immature granulomas, regardless of the time point analyzed (Fig. 3). Distribution of F4/80+ cells within granulomas was characterized by a strong signal in Kupffer cells situated at the periphery of granulomas and a lighter signal in fused Kupffer cells containing *L. donovani* amastigotes in the centre of the granuloma (Fig. 3).

Immunohistochemical staining for iNOS also showed a higher mean percentage staining in immature granulomas, regardless of the time point. As occurred for F4/80+ immunostaining, there was a decrease in the expression of iNOS as the granuloma categories evolved within the same time point. However, compared with F4/80+ cells, the highest expression of iNOS was observed at 35 dpi and not at 63 dpi. In fact, there was a statistically significant reduction in the expression of iNOS for the three granuloma categories at 63 dpi, when compared to 35 dpi (immature: *t* = 4.589, *P* < 0.001; mature: *t* = 10.520, *P* < 0.001; clear: *t* = 8.653, *P* < 0.001) (Fig. 3). Positive staining for iNOS was mainly observed in the cytoplasm of fused Kupffer cells in the centre of the granuloma and was especially marked in immature granulomas (Fig. 3).

#### T lymphocytes (CD3+)

**Fig. 4:**
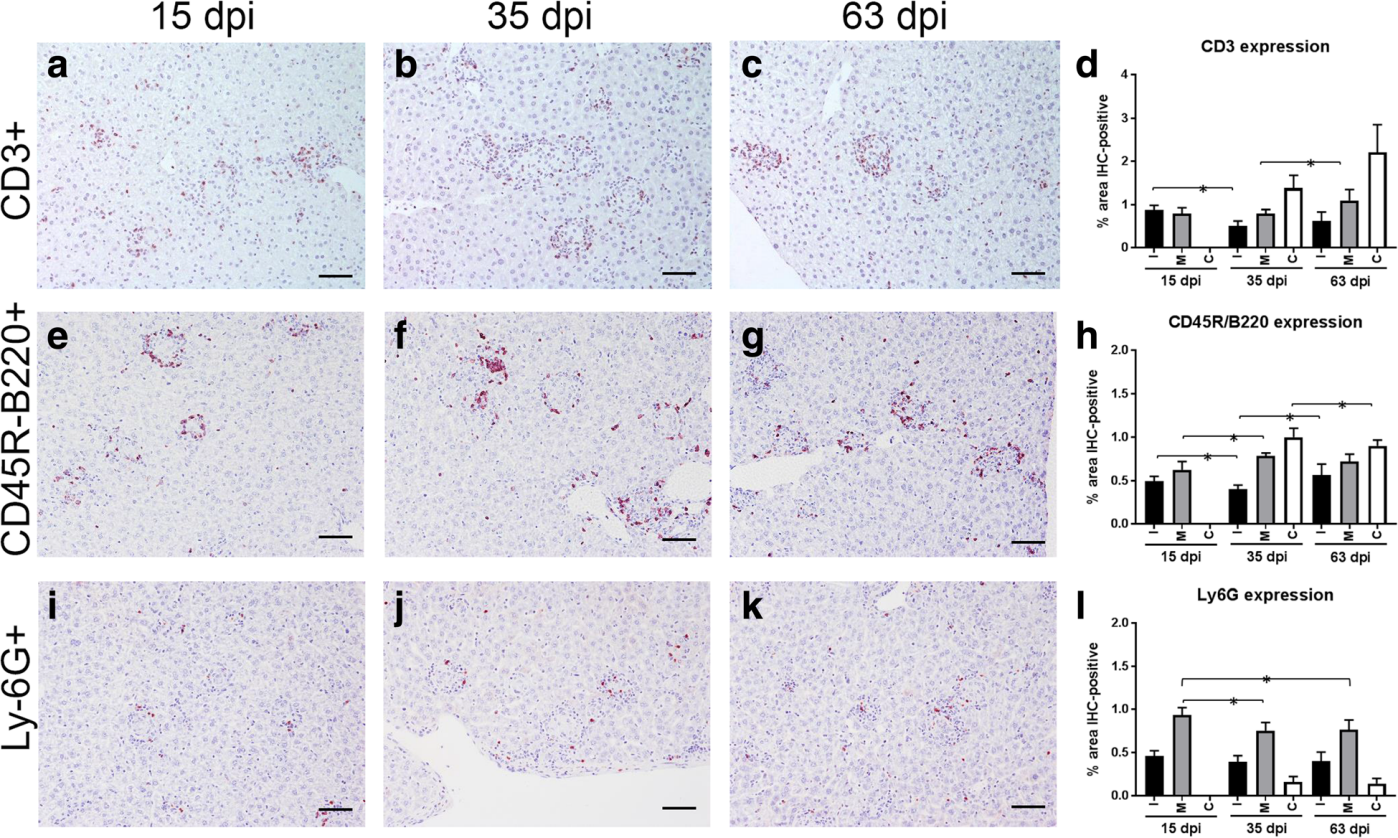
Immunohistochemical detection of CD3+ T cells within hepatic granulomas at 15 dpi (**a**), 35 dpi (**b**) and 63 (**c**) dpi. **d** Percentage of immunostained area for CD3 at 15, 35 and 63 dpi within immature (I), mature (M) and clear (C) granulomas. Immunohistochemical detection of CD45R/B220+ cells (B cells) at 15 dpi (**e**), 35 dpi (**f**) and 63 dpi (**g**). **h** Percentage of immunostained area for CD45R/B220 at 15, 35 and 63 dpi within immature (I), mature (M) and clear (C) granulomas. Immunohistochemical detection of Ly6G+ cells (neutrophils) at 15 dpi (**i**), 35 dpi (**j**) and 63 dpi (**k**). **l** Percentage of immunostained area for Ly6G at 15, 35 and 63 dpi within immature (I), mature (M) and clear (C) granulomas. *P < 0.05. *Scale-bars*: 50 μm

An increase in the expression of CD3 was noted as granuloma categories evolved in animals at 35 and 63 dpi, while a reduction was observed at 15 dpi. The highest expression of CD3 was observed in animals euthanized at 63 dpi. The only statistically significant differences were observed when comparing mature granulomas between animals euthanized at 35 and 63 dpi (*t* = -2.071, *P* < 0.001) and immature granulomas at 15 and 35 dpi (*t* = 3.507, *P* < 0.001) (Fig. 4). Distribution of CD3+ cells was similar within granuloma categories and time points. CD3+ cells were dispersed in the cellular infiltrate surrounding the fused Kupffer cells situated in the granuloma centre (Fig. 4).

#### B lymphocytes (CD45R-B220+)

Immunohistochemical staining for CD45R-B220 showed the location of B lymphocytes within the granuloma. An increase in the expression of CD45R-B220 was observed as granuloma categories evolved within the same time point (Fig. 4). When comparing granulomas from mice euthanized at 15 and 35 dpi, a statistically significant reduction in the expression of CD45R-B220 was observed for immature granulomas (*t* = 2.029, *P* < 0.043), while a statistically significant increase was observed for mature granulomas (*t* = -2.213, *P* < 0.027). The expression of CD45R-B220 in mature granulomas was quite homogeneous when comparing mice euthanized at 35 and 63 dpi (Fig. 4). Most of the cells shaping the cellular infiltrate surrounding the fused Kupffer cells were CD45R-B220+ cells when compared with CD3+ cells (Fig. 4).

#### Neutrophils (Ly-6G+)

The expression of Ly-6G within granulomas was higher in mature compared to immature and clear granulomas. Within mature granulomas a significantly higher expression of Ly-6G was observed at 15 dpi when compared to 35 dpi (*t* = 3.019, *P* < 0.043) and 63 dpi (*t* = 4.034, *P* < 0.035). In clear granulomas, neutrophils were only observed occasionally. The expression of Ly-6G was generally lower compared to the other studied cell markers, particularly at later time points (Fig. 4).

## Discussion

The histopathological hallmark of hepatic resistance to visceralizing species of *Leishmania* is the development of functional granulomas [10, 11, 14]. Here we used IHC as a tool to characterise the different cells involved in the hepatic granuloma development at different stages of experimental VL in BALB/c mice, complemented by quantitative image analysis and detection of *Leishmania* antigen.

Immunohistochemical methods described here are a powerful tool to characterise host responses to infection in situ in mouse models of *Leishmania* spp. infection. These have also been used in other granulomatous diseases that share some, though not all properties with VL [15–18]. Additionally these techniques can be utilised to evaluate the cellular mechanism of new vaccines, drugs and treatment regimens [19–21]. Therefore this study provides in depth insights into the cellular dynamics of experimental VL in a context relevant to advancing health.

In agreement with previous studies [22, 23], we observed that the majority of hepatic granulomas at 15 dpi were immature, with a heavy presence of amastigotes. Early amastigote replication in tissue macrophages is regulated by the phagosomal proton-cation antiporter encoded by the *Slc11a1* gene (formerly *Nramp1*), and high amastigote loads in the livers of BALB/c mice have been linked to mutations in the *Slc11a1* gene [24–27]. Control of hepatic parasite growth at later stages of infection corresponds to the development of acquired immune mechanisms [27, 28]. The increase in the percentage of mature granulomas and the appearance of clear granulomas at 35 and 63 dpi reflect the onset of acquired immune responses and disease control, as the leishmanicidal efficacy of hepatic granulomas is dependent on their degree of maturation [23, 29]. In this sense, it has been proposed that determining the degree of maturation of hepatic granulomas constitutes an effective tool for selecting VL vaccine candidates [30].

We also observed significant differences in hepatic granuloma sizes between categories, possibly linked to differences in the cellular composition of respective granulomas in each category. Immature granulomas are shaped by the presence of phagocytic cells, whereas mature granulomas contain higher numbers of lymphocytes, as observed by immunohistochemical detection of T and B cells. Clear granulomas are smaller than mature granulomas and contain less F4/80+ cells.

The highest expression of F4/80 found in immature granulomas and the decrease in the expression of F4/80 observed as the granuloma category evolved within the same time point, reflect that resident macrophages are the first line of defence against *Leishmania* parasites within this tissue. Moreover, monocytes are recruited into the granuloma by chemokines (CCL3, CCL2 and CXCL10) secreted by Kupffer cells infected with the parasite [27].

Similarly to F4/80+ cells, immunohistochemical staining for iNOS showed a higher mean percentage staining in immature granulomas regardless of the time point, and there was a decrease in the expression of iNOS as the granuloma categories evolved within the same time point. Hepatic resistance against *L. donovani* infection correlates well with the generation of reactive oxygen and reactive nitrogen intermediates [12]. Macrophages are the main producers of iNOS, especially when activated by the intervention of T lymphocytes. Nitric oxide produced by iNOS is believed to be of prime importance in the cure of murine leishmaniasis, and in activating murine macrophages to kill intracellular parasites [31]. This has been illustrated by studies of iNOS gene knockout mice, which do not self-resolve infection [32]. The statistically significant reduction in the expression of iNOS for the three granuloma categories at 63 dpi, when compared to 35 dpi, may be related to the presence of lower numbers of *Leishmania* amastigotes in the liver at this phase of infection. The course of infection in BALB/c mice infected with the *L. donovani* strain used here is well documented [33, 34]. An increase in hepatic parasite burden is observed until amastigote growth is controlled around 28 dpi, followed by clearance of parasites at later time points. In our study, complete absence of *Leishmania* amastigotes was not observed by 63 dpi. It has previously been reported that sterile immunity in the liver is not achieved, but the presence of a residual parasite population is thought to incite a small but enduring immune response that provides long-term immunity to reinfection [27].

Expression of CD3 was lower at 15 dpi and increased as granuloma categories evolved at 35 and 63 dpi. The highest expression of CD3 was observed at the last time point, which is in agreement with the continuous maturation of hepatic granulomas. Gene knockout and antibody neutralization studies showed that granuloma maturation in the liver of infected mice requires CD4 and CD8 T cells and pro-inflammatory cytokines such as IL-12, IFN-g and IL-2 to be functional [29, 35, 36].

Most of the cells that shaped the cellular infiltrate surrounding the fused Kupffer cells were CD45R-B220+ cells and the expression of this marker increased as the granuloma categories evolved within each time point. B cells have previously been observed in hepatic granulomas of *L. donovani* infected BALB/c mice [35] and have been shown to accumulate in hepatic granulomas in infected mice over time, exhibiting a highly motile behaviour [37]. They play a role in preventing liver pathology via control of neutrophil infiltration [38]. The increase of CD45R-B220+ cells over time is also related to the establishment of acquired immunity during the maturation process of granulomas [14].

The number of Ly6G+ neutrophils within the hepatic granulomas was low and this cell population was mainly observed in mature granulomas at all time points. Previous studies have demonstrated protective effects of neutrophils in livers of *L. donovani* infected mice and small numbers of infiltrating neutrophils have been observed in granulomas at 14 dpi [38, 39]. A contribution of neutrophils to the maturation of functional hepatic granulomas and induction of hepatic iNOS has been suggested [39].

## Conclusions

We observed that hepatic lesions start as a basic fusion of Kupffer cells, producing high levels of iNOS, and move to a more complex granuloma, related to an adaptive immune response with the presence of higher numbers of B and T lymphocytes and a final resolution towards collagen deposition. The analyses described here, based on infection with an established laboratory strain of *L. donovani*, can be applied to other host-parasite combinations, including genetically manipulated parasites and recent clinical isolates. The panel of cellular markers can be extended to include other, less-studied cell types. Paraffin blocks from this study have been archived and are available for future analysis.

## Abbreviations

CL: Cutaneous leishmaniasis
dpi: Days post-infection
H&E: Hematoxylin and eosin
IHC: Immunohistochemistry
iNOS: Inducible nitric oxide synthase
LDU: Leishman-Donovan units
NTD: Neglected tropical disease
ROI: Region of interest
SD: Standard deviation
VL: Visceral leishmaniasis
